# Loss of mitochondrial energetics is associated with poor recovery of muscle function but not mass following disuse atrophy

**DOI:** 10.1152/ajpendo.00161.2019

**Published:** 2019-09-03

**Authors:** Michelle B. Trevino, Xiaolei Zhang, Robert A. Standley, Miao Wang, Xianlin Han, Felipe C. G. Reis, Muthu Periasamy, Gongxin Yu, Daniel P. Kelly, Bret H. Goodpaster, Rick B. Vega, Paul M. Coen

**Affiliations:** ^1^Sanford Burnham Prebys Medical Discovery Institute at Lake Nona, Orlando, Florida; ^2^Translational Research Institute for Metabolism and Diabetes, AdventHealth, Orlando, Florida; ^3^Cardiovascular Research Institute and Department of Medicine, Perelman School of Medicine, University of Pennsylvania, Philadelphia, Pennsylvania

**Keywords:** cardiolipin, mitochondria, muscle atrophy, PGC-1

## Abstract

Skeletal muscle atrophy is a clinically important outcome of disuse because of injury, immobilization, or bed rest. Disuse atrophy is accompanied by mitochondrial dysfunction, which likely contributes to activation of the muscle atrophy program. However, the linkage of muscle mass and mitochondrial energetics during disuse atrophy and its recovery is incompletely understood. Transcriptomic analysis of muscle biopsies from healthy older adults subject to complete bed rest revealed marked inhibition of mitochondrial energy metabolic pathways. To determine the temporal sequence of muscle atrophy and changes in intramyocellular lipid and mitochondrial energetics, we conducted a time course of hind limb unloading-induced atrophy in adult mice. Mitochondrial respiration and calcium retention capacity were diminished, whereas H_2_O_2_ emission was increased within 3 days of unloading before significant muscle atrophy. These changes were associated with a decrease in total cardiolipin and profound changes in remodeled cardiolipin species. Hind limb unloading performed in muscle-specific peroxisome proliferator-activated receptor-γ coactivator-1α/β knockout mice, a model of mitochondrial dysfunction, did not affect muscle atrophy but impacted muscle function. These data suggest early mitochondrial remodeling affects muscle function but not mass during disuse atrophy. Early alterations in mitochondrial energetics and lipid remodeling may represent novel targets to prevent muscle functional impairment caused by disuse and to enhance recovery from periods of muscle atrophy.

## INTRODUCTION

Skeletal muscle atrophy occurs during prolonged contractile inactivity because of immobilization, injury, or bed rest (29, 40). The resulting functional impairment, increased risk of falls and fractures, insulin resistance, and loss of cardiovascular fitness are serious health issues, particularly for older adults. Prolonged disuse can result in a loss of ~0.5%–0.6% of skeletal mass per day (62). A complete recovery of muscle mass and strength following a period of disuse is also an important therapeutic goal.

Muscle mass is regulated by a fine balance between protein synthesis and degradation pathways (18). The mechanisms responsible for atrophy are complex but include decreased anabolic signaling and increased protein degradation (ubiquitin-mediated pathways) (4). The insulin-like growth factor 1 (IGF-1) pathway is a key mediator of muscle growth (18). Impaired activation of the phosphatidylinositol 3-kinase-Akt-mammalian target of rapamycin (mTOR) signaling pathway results in decreased protein synthesis and myocyte atrophy (6, 48). Protein degradation is mediated primarily by the autophagy/lysosome system and the ubiquitin-proteasome pathway via key ubiquitin ligases, including muscle ring finger 1 (MuRF1) and muscle atrophy F-box (MaFbx or atrogin-1) (5, 23). Expression of *Trim63* and *Fbxo32* (the genes encoding MuRF1 and atrogin-1, respectively) is in large part regulated by the FoxO1 and FoxO3 transcription factor (52, 57). FoxO transcription factors are inactivated by direct phosphorylation by Akt providing a regulatory link to the IGF-1 pathway. Indeed, the IGF-1/phosphatidylinositol 3-kinase/Akt pathway has been shown to directly antagonize FoxO-mediated activation of MuRF1 and atrogin-1 thereby tipping the balance toward growth and hypertrophy (50, 57).

A link between mitochondria dysfunction and muscle atrophy has been known for 40 yr (10). Mitochondrial oxidative stress stimulates muscle protein breakdown by increasing the expression of proteins involved in the proteolytic degradation pathways (autophagy and proteasome system) (1, 15, 28, 36, 38). Oxidatively modified proteins are also more susceptible to proteolytic degradation by the 20S proteasome (25, 55). Oxidative stress can also depress protein synthesis by decreasing phosphorylation of 4E binding protein 1 and impairing mTOR assembly, thereby halting mRNA translation at the level of initiation (43, 53, 69). Therefore, the decrease in muscle protein synthesis that occurs from disuse could be linked to increased production of reactive oxygen species that occurs in inactive muscle. Energetic stress (reduced ATP/AMP ratio) may also activate the AMP kinase-FoxO3 pathways (46, 47). Activation of AMP kinase because of energetic stress results in the activation of the transcription factor FoxO3. This results in increased expression of atrogin-1, MuRF1, light chain (LC) 3, and Bnip3 (24, 46), key proteins involved in the ubiquitin-proteasome system and lysosome-autophagy system. In support of the link between mitochondria and muscle mass, genetic and biochemical approaches to preserve mitochondrial function during disuse atrophy also preserve muscle mass. Transgenic overexpression of the peroxisome proliferator-activated receptor (PPAR)-γ coactivator-1α (PGC-1α) and treatment with mitochondrial-targeted antioxidants preserved muscle mass during hind limb unloading. However, the temporal changes in muscle mass and function have not been investigated (7, 9, 20, 31, 51).

To better understand the time course and mechanisms involved in disuse atrophy, we initially performed unbiased transcriptomics using RNA sequencing (RNA-Seq) of muscle biopsies from older individuals subjected to complete bed rest. This analysis revealed a marked inhibition of nuclear-encoded mitochondrial transcripts regulated by PGC-1α and downstream effectors, such as the PPAR and the estrogen-related receptor (ERR). Next, we utilized a mouse hind limb-unloading model to examine temporal changes in mitochondrial energetics and key aspects of the atrophy program during muscle atrophy. A decline in mitochondrial function, independent of change in markers of content, was evident before a significant loss of muscle mass along with remodeling of cardiolipin species. Finally, we demonstrate that skeletal muscle-specific deletion of PGC-1α/β did not affect disuse atrophy caused by unloading. Instead, we observed an impaired functional recovery following reloading. These results suggest that therapeutic approaches aimed at mitochondrial function could prove useful to enhance recovery from muscle atrophy.

## MATERIALS AND METHODS

### 

#### Bed rest study design.

A group of older adults were enrolled and completed 10 days of bed rest (*n* = 3, 60–76 yr old). These individuals were a subgroup from a larger investigation of the effects of bed rest (16, 56). A detailed presentation of the subject characteristics, study design, inclusion and exclusion criteria, supplementation, compliance, bed rest, body composition, strength testing, and resistance-exercise training measurements and related findings have been reported previously (16). The study was conducted at the University of Arkansas for Medical Sciences clinical research center, Little Rock, AR, and approved by the Institutional Review Board of the University of Arkansas for Medical Sciences. All study procedures, risks, and benefits were explained to the subjects before they gave written consent to participate.

#### Percutaneous muscle biopsy.

Participants underwent a muscle biopsy of the medial vastus lateralis before and on the last day of bed rest. A biopsy sample was taken 10–15 cm above the knee under local anesthesia (1% lidocaine HCl) with a 5-mm Bergstrom needle with suction as previously described (44). Biopsy specimens were flash frozen in liquid N_2_ and stored at −80°C.

#### Animals.

All animal experiments and euthanasia protocols were conducted in strict accordance with the National Institutes of Health (NIH) guidelines for humane treatment of animals and approved by the Institutional Animal Care and Use Committee at the Sanford Burnham Prebys Medical Discovery Institute at Lake Nona (protocol no. 2013–0102). Eight-week-old C57BL/6J mice and PGC-1α floxed mice were obtained from Jackson Laboratory (Bar Harbor, ME). Skeletal muscle-specific PGC-1α/β knockout mice were produced by crossing mice harboring Cre recombinase under control of the myogenin promoter (35) (a kind gift from Dr. Eric Olson, UT Southwestern) to mice carrying floxed alleles of PGC-1α (37) and PGC-1β (33). These mice were originally on the C57BL/6J background and then crossed to C56BL/6N mice harboring the wild-type (WT) *NNT* gene. All muscle-specific PGC-1α/β knockout and control mice used in the hind limb-unloading experiments were *NNT* WT genotype. PGC-1α/β floxed mice that were Cre^-^ were used as WT controls. Hind limb unloading and reloading studies with the skeletal muscle PGC-1α/β knockout were performed with 12-wk-old female mice. All mice were fed ad libitum with a standard chow diet (cat. no. 2916, Harlan-Teklad, Houston, TX) and water/hydrogel (ClearH_2_O, Westbrook, ME) and housed at 22°C with a 12-h light/dark cycle.

#### Tail suspension hind limb unloading.

Hind limb suspension (non-weight bearing) was achieved by subjecting mice to tail suspension as a commonly used animal model of muscle disuse atrophy. Prior to tail suspension experiments, the individual mice acclimated to single-cage housing and routine handling for 3 days. Tail suspension was performed using a modified version of the Morey-Holton and Globus protocol (22). Briefly, a small metallic hook was taped to the base of the tail using nonabrasive adhesive tape wrapped in a helical pattern. The hook was then attached to a small swivel key chain that was attached to a metal rod that ran the length of the microisolator cage. The mice were free to move on a *y*-axis and rotate 360 degrees and had access to all areas of the cage. The hind limbs were maintained just off the cage floor with the body of the mouse at a ~30° angle from the cage floor. Control mice were separated into the individual cages with exactly the same conditions as the unloading groups, but without tail suspension. Reloading was performed by removing the hook from the tail and allowing free movement in the cage as with control mice. Mice were reloaded for 7 or 21 days as indicated. At the end of each experimental protocol and indicated time point, the mice were fasted for 4 h and euthanized by CO_2_ asphyxiation and cervical dislocation. The hind limb muscles, including soleus, gastrocnemius, and quadriceps femoris muscle groups, were immediately harvested and weighed. The soleus muscle from the right hind limb was used for fresh tissue assay immediately and other muscles were snap frozen in liquid N_2_ for other analysis.

#### Myofiber bundle preparation.

Permeabilized fiber bundles (1–3 mg) were prepared immediately following tissue harvest, as previously described (12, 13). Briefly, myofibers from soleus were gently teased apart into small bundles using fine-nosed tweezers while submerged in ice-cold biopsy preservation solution (7.23 mM K_2_EGTA, 2.77 mM Ca K_2_EGTA, 20 mM imidazole, 0.5 mM DTT, 20 mM taurine, 5.7 mM ATP, 14.3 mM phosphocreatine, 6.56 mM MgCl_2_-6H_2_O, 50 mM MES (not hydrate), and pH was adjusted to 7.1 by 5N KOH) in a petri dish. For respiration and H_2_O_2_ emission assays, the fiber bundles were permeabilized in saponin (2 mL of 50 μg/mL) for 20 min on ice by shaker and then washed twice in Buffer Z (105 mM K-MES, 30 mM KCl, 10 mM, KH_2_PO_4_, 5 mM MgCl_2_-6H_2_O, 5 mg/mL BSA, 1 mM EGTA, pH 7.4 with KOH) with blebbistatin (25 μM) to inhibit contraction. For the calcium uptake assay, the fiber bundles were washed twice in Buffer Y (250 mM sucrose, 10 mM Tris-HCl, 20 mM Tris base, 10 mM KH_2_PO4, and 0.5 mg/mL BSA) following saponin permeabilization.

#### Mitochondrial respiration.

Respirometry assays were conducted using an Oxygraph-2k (Oroboros Instruments, Innsbruck, Austria). The myofiber bundles were gently placed into the respirometer chambers, and after a stable baseline was reached, the assay protocol was run in duplicate at 37°C and between 350 and 200 nmol of O_2_ in Buffer Z with blebbistatin (25 μM). Complex I-supported LEAK (State 4) respiration was determined through the addition of glutamate (5 mM) and malate (2 mM). ADP (4 mM) was added to elicit complex I supported oxidative phosphorylation (OXPHOS; P_CI_ or State 3) respiration. Succinate (10 mM) was then added to elicit complex I & II-supported OXPHOS (P_CI+CII_) respiration. Cytochrome c (10 μM) was added to assess the integrity of the outer mitochondrial membrane. If the addition of cytochrome c resulted in a >10% increase in respiration, the assay data were not included in the final analysis. Oxygen flux was normalized and expressed as pmol·s^−1^·mg^−1^ wet wt of muscle fiber.

#### Mitochondrial calcium retention capacity.

A continuous spectrophotometric assay was utilized to measure mitochondrial calcium retention capacity within soleus fiber bundles. The response of the mitochondria to addition of calcium depends on the amount and the level of mitochondrial calcium added. Muscle fibers were exposed to progressive calcium (20–60 μM) loading in the presence of 5 mM malate, 10 mM glutamate, 10 mM succinate, 0.025 mM ADP, and 0.002 mM thapsigargin to inhibit calcium uptake via the sarco/endoplasmic reticulum Ca^2+^-ATPase (SERCA). Calcium Green (Invitrogen, Grand Island, NY) at 1 μM was employed to monitor changes in extramitochondrial calcium concentration. All experiments were run at 37°C in Buffer Y containing 2 U/mL hexokinase and 5 mM 2-deoxyglucose and 25 μM blebbistatin using Luminescence Spectrometer (LS50B, Perkin Elmer, Waltham, MA). The spectrometer readings were performed at excitation/emission of 506/532 nm. At the conclusion of each experiment, muscle was dried and weighed, and total calcium uptake was normalized to dry weight of muscle fiber.

#### Lipidomics.

Multidimensional mass spectrometry-based shotgun lipidomics was employed to measure and characterize the lipid patterns in mouse soleus muscle. Briefly, the lipids were extracted from soleus muscle by a modified Bligh and Dyer procedure with LiCl solution (65). The lipid extracts were finally flushed with nitrogen, capped, and stored at −20°C for electrospray ionization mass spectrometry. By incubating at 37°C for 24 h, 4-hydroxyalkenal species were measured by derivatization with 125 mM carnosine (63). The derivatization of phosphatidylcholine was performed by incubating with 4 mM Fmol-Cl and 8 mM 4-dimethylaminopyridine for 2 h (27). Diacylglycerols were characterized by derivatization with N,N-dimethylglycine (66). Nonesterized fatty acid species were measured by incubating with N-[4-(aminomethyl) phenyl] pyridinium (64). Internal standards were added before the extraction for normalization. For electrospray ionization direct infusion analysis, lipid extract was further diluted to a final concentration of ~500 fmol/μL by CHCl_3_/MeOH/isopropanol (1/2/4, vol/vol/vol) with or without 0.02% (vol/vol) LiOH-saturated MeOH solution, and the mass spectrometric analysis was performed on a triple quadrupole mass spectrometer (Thermo TSQ VANTAGE; Thermo, San Jose, CA) or a Q-Exactive mass spectrometer (Thermo) equipped with an automated nanospray device (TriVersa NanoMate, Advion Bioscience, Ithaca, NY) and operated with Xcalibur software. Data processing of mass spectrometry analyses, including ion peak selection, data transferring, baseline correction, deisotoping, peak intensity comparison, and quantification, is conducted by self-programmed Microsoft Excel macros (67). Data from mouse soleus muscle samples were normalized to protein content.

#### RNA isolation and quantitative RT-PCR.

Tissue homogenates of the soleus muscle (~5–10 mg) were generated by suspending the tissue in QIAzol lysis reagent and homogenized using the high-throughput Precellys bead-homogenization system (Bertin Instruments, France). High-quality total RNA was prepared using the Qiagen RNeasy kit (Qiagen, Valencia, CA) and transcribed to cDNA using the AffinityScript quantitative PCR cDNA synthesis kit (Agilent Technologies, Santa Clara, CA). Gene expression was measured with the Roche LightCycler 480 Real Time PCR system (Roche, Indianapolis, IN). Arbitrary units of target mRNA were corrected to the expression of 36b4 (Rplp0).

#### RNA-Seq and informatics.

Total RNA was isolated using the RNEasy Fibrous Tissue Kit (Qiagen). The quality of total RNA was assessed by the Agilent Bioanalyzer Nano Chip (Agilent Technologies). As starting material, 1 μg of total RNA was used to construct the RNA-Seq library using the Truseq Stranded Total RNA Library preparation kit (Illumina Incorporated) per the manufacturer’s instructions. Briefly, the total RNA is ribo-depleted to remove rRNA. The remaining non-rRNA is fragmented into small pieces using divalent cations under elevated temperatures. Following fragmentation, the first-strand cDNA was synthesized using random primers and followed by second-strand synthesis using DNA Polymerase I. The cDNA was then ligated with index adapters for each sample, followed by purification, and was then enriched with PCR to create the final library. The quality and quantity of the libraries were detected by Agilent Bioanalyzer and Kapa Biosystems quantitative PCR. Multiplexed libraries are pooled, and single-end 50-bp sequencing was performed on one flow cell of an Illumina Hiseq 2500. Reads from 36 samples were mapped to a mouse genome, mm10, using TopHat 2.0.1 (https://ccb.jhu.edu/software/tophat/index.shtml); mapped reads were filtered based on the mapping quality. The overall mapping rates were ~90%. mRNA quantification and normalization were done using Partek Genomics Suite 6.6 annotation database using RefSeq Transcript 77 (July 2016). R package DeSeq (http://bioconductor.org/packages/release/bioc/html/DESeq.html) was used to analyze the differential expression of mRNAs. Transcripts with reads per kilobase million (RPKM) >1 and an uncorrected *P* value < 0.5 were used for subsequent pathway and upstream regulator analysis. The principal component analysis (PCA) follows the processing pipeline as described (14), with some modifications. Briefly, transcripts with an RPKM value < 0.5 were removed, then log_2_ transformed and quantile normalized. The PCA was then performed for clustering analysis and feature evaluation (34).

#### Gene ontology, Kyoto Encyclopedia of Genes and Genomes pathway, and upstream regulator analysis.

The Database for Annotation, Visualization and Integrated Discovery v6.8 was used to identify enriched gene ontology (GO) and Kyoto Encyclopedia of Genes and Genomes (KEGG) pathways. Ingenuity Pathway Analysis Upstream Regulator was used to infer and score regulator networks upstream of gene expression data to identify putative upstream transcriptional regulators (32). The gene expression data discussed in this publication have been deposited in National Center for Biotechnology Information’s Gene Expression Omnibus and are accessible through Gene Expression Omnibus series accession no. GSE126865.

#### Immunoblot analysis.

Frozen soleus was mechanically homogenized (PowerGen 125, Fisher Scientific, Hampton, NH) and sonicated with 50% duty cycle (Branson Sonifier 450) in ice-cold cell lysis buffer (cat. no. 9803S, Cell Signaling Technology, Danvers, MA), including a protease inhibitor cocktail (Roche Diagnostics GmbH, Mannheim, Germany). Total protein in each sample was determined using the bicinchoninic acid assay (Thermo Scientific, Rockford, IL). Samples were separated on the 8%–12% SDS-PAGE gel followed by transfer onto polyvinylidine difluoride membranes (Bio-Rad Laboratories, Hercules, CA). Membranes were blocked in 5% nonfat milk and incubated with the following primary antibodies. Antibodies directed against Foxo3a (cat. no. ab47409), pFOXO3a (cat. no. ab31109), Atrogin-1 (FBX32, ab17249), Pink1 (cat. no. ab23707) and total OXPHOS cocktail (cat. no. ab110413) antibodies were purchased from Abcam (Cambridge, MA). LC3B (cat. no. 3868), S6 (cat. no. 2217), pS6 (cat. no. 2215), phospho-Akt (Ser^473^, cat. no. 9273), Akt (cat. no. 9272), mTOR (cat. no. 2972), phospho-mTOR (Ser^2448^, cat. no. 2971), and α-tublin (cat. no. 2125S) antibodies were from Cell Signaling (Danvers, MA). Membranes were then incubated in appropriate species-specific secondary antibodies and developed with X-ray films. Protein loading was controlled by normalizing bands of interest to α-tubulin. The immunoblotting band was quantified by Image J (National Institutes of Health, Bethesda, MD).

#### Ex vivo muscle function.

Ex vivo muscle contractility measurements were performed as previously described (17, 41). Briefly, soleus muscle was harvested following 21 days of reloading in the muscle-specific PGC-1α/β knockout and WT control mice. The muscle was excised and pinned to the silicone base of a 10-cm dish containing O_2_-perfused Krebs buffer. Looped sutures tied around each end of the tendons were used to mount the soleus between a force transducer (model 6650LR, Aurora Scientific) in a glass bath containing O_2_-perfused Krebs buffer at 22°C. Next, optimum twitch length was determined by incrementally lengthening the muscle until gains in twitch force reached a plateau. Force frequency and fatigability were tested using a dual-mode muscle lever system (model 300A, Aurora Scientific) controlled by 610A Dynamic Muscle Control Software v5.415. The force-frequency relationship was assessed using the 1200A isolated muscle test system (Aurora Scientific). A force-frequency protocol was followed using the following frequencies: 1, 15, 30, 50, 80, 120, 150, and 200 Hz, waiting 30, 30, 60, 90, 120, 120, 120, and 120 s, respectively, between each 300-ms activation. To induce fatigue, the muscle was stimulated with 300-ms pulses at 40 Hz with a rest interval of 2 s for 5 min (total of 150 stimuli) after maximal force protocol was completed. Muscle-specific forces, physiological cross-sectional area, and fatigue were analyzed as described previously (42).

#### Statistical analysis.

All data are represented as the mean ± SE. All the statistical analyses were performed by GraphPad Prism. The differences between groups were conducted using ANOVA or *t* test (paired and unpaired) approaches with Tukey’s multiple comparison test. *P* < 0.05 was considered significant.

## RESULTS

### 

#### Disuse atrophy results in marked inhibition of mitochondrial energy metabolic pathways.

**Fig. 1.**
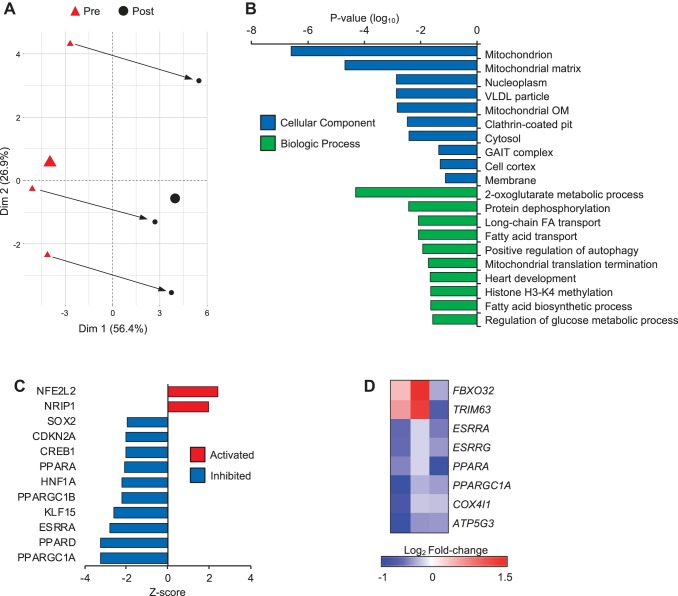
Inhibition of mitochondrial energy metabolic pathways occurs in human disuse atrophy. *A*: a principal component analysis (PCA) plot was generated for the RNA-sequencing data set from the three subjects used in this analysis. The Pre (red triangle) and Post (closed circle) time points are indicated and connected by an arrow for each subject. The large symbols indicate the average for all three subjects at each time point. *B*: the 10 most significant gene ontology cellular component (blue bars) and biologic process (green bars) terms using transcripts significantly downregulated during bed rest are shown. *C*: Ingenuity upstream regulator analysis tool was used to predict activity of transcription factors that were affected by bed rest using all up- or downregulated transcripts with reads per kilobase million > 1 and *P* < 0.05. Transcription factors predicted to be activated are shown in red (*z*-score >2) and inhibited are shown in blue (*z*-score <−2). *D*: the heat map represents the log_2_ fold-change in gene expression for the indicated transcript during bed rest for each individual subject. HNF1A, hepatocyte nuclear factor 1A; KLF, Krüppel-like factor; PPAR, peroxisome proliferator-activated receptor.

To identify pathways involved in disuse muscle atrophy, we performed RNA-Seq of muscle biopsy samples from older individuals before and after 10 days of complete bed rest (16, 56). The characteristics of the subjects used for this analysis are shown in Table 1. Interestingly, one subject did not show significant atrophy. However, all subjects displayed diminished muscle strength following the bed rest period. A PCA of the RNA-Seq data showed that bed rest had a profound impact on the skeletal muscle transcriptome (Fig. 1*A*). As a first step to identify pathways affected during bed rest, significantly up- or downregulated transcripts (RPKM >1 and *P* < 0.05) were used for unbiased functional annotation analysis. As expected, KEGG terms for the proteasome and ubiquitin-mediated proteolysis were identified using transcripts upregulated following bed rest, whereas PPAR signaling was the top term using downregulated transcripts (Supplemental Fig. S1*A*; all Supplemental Material is available online at https://doi.org/10.6084/m9.figshare.8021150.v5). KEGG terms for TCA cycle and carbon metabolism were also enriched using downregulated transcripts, suggesting inhibition of energy metabolism with bed rest. Supportive of this notion, when downregulated transcripts were examined, GO cellular component terms related to mitochondria were strongly represented (Fig. 1*B*). Further analysis of GO terms related to biologic processes revealed pathways related to the TCA cycle and fatty acid transport were downregulated with bed rest (Fig. 1*B*). These pathways contained transcripts representative of energy metabolic pathways, including fatty acid oxidation (FAO), the TCA cycle, and the electron transport chain and OXPHOS (data not shown). To determine regulatory networks involved in these changes, we used Ingenuity upstream regulator analysis to predict changes of transcription factor activity with bed rest. All up- and downregulated transcripts were used in this analysis. As shown in Fig. 1*C*, the nuclear receptors and ligand-binding transcriptional predicted to be the most significantly inhibited included PGC-1α and PGC-1β (*PPARGC1A*, *PPARGC1B*), PPARα/PPARδ (*PPARA*, *PPARD*), Krüppel-like factor 15, and ERRα (*ESRRA*). These transcription factors have been shown to be key nodal regulators of mitochondrial biogenesis and energy metabolism, including FAO (60). Interestingly, this analysis also predicts activation of RIP140 (*NRIP1*), a factor known to function as an inhibitor of these factors and pathways (Fig. 1*C*) (19). The mRNA level of these transcription factors was next measured to determine if the regulation of these pathways was due to changes in the expression level of these regulatory factors. Although not statistically significant because of small sample size (*n* = 3), levels of the E3 ubiquitin ligases, atrogin-1 (*FBXO32*) and MuRF1 (*TRIM63*) were elevated (Fig. 1*D* and Supplemental Fig. S1*B* https://doi.org/10.6084/m9.figshare.8021150.v5). The expression of ERRα/γ, PPARα, and PGC-1α were all reduced in all three subjects examined following bed rest (Fig. 1*D* and Supplemental Fig. S1*B* https://doi.org/10.6084/m9.figshare.8021150.v5). This was accompanied by reductions in the expression of representative gene targets regulated by these factors (*COX4I1* and *ATP5G3*) (Fig. 1*D* and Supplemental Fig. S1*B*).

**Table 1.** Subject characteristics

	*Subject 1*		*Subject 2*		*Subject 3*	
	Pre	Post	Pre	Post	Pre	Post
Age, yr	64		70		76	
Body weight, kg	83.1	79.7	73.9	74.6	80.9	80.3
Total body fat, kg	26.68	26.55	29.67	29.32	34.94	38.32
Total lean mass, kg	52.76	48.55	40.55	41.31	43.1	38.73
Leg lean mass, kg	15.69	14.27	11.85	12.09	14.2	11.41
Knee extensor 60°, Nm	219.7	166	148	128.3	120	98.7

#### Changes in mitochondrial content and function occur early with disuse atrophy.

**Fig. 2.**
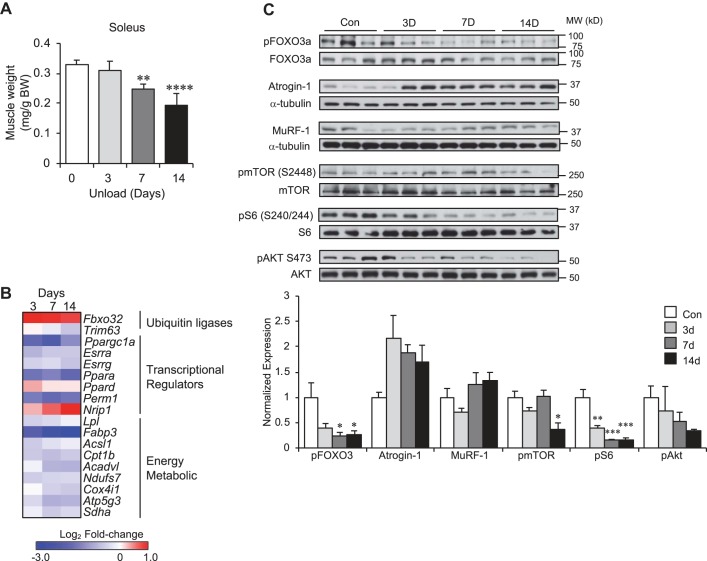
Inhibition of mitochondrial energy pathways occurs early in disuse atrophy. *A*: soleus muscle mass is shown at various time points following hind limb unloading. Bars represent mean ± standard deviation (*n* = 5 per group). *B*: heat map represents log_2_ fold-change of indicated transcript at 3, 7, and 14 days (d) of hind limb unloading (*n* = 5 per time point). *C*: Western blot analysis was performed of various signaling pathways and other markers of atrophy in soleus muscle of control 3, 7, and 14 days of hind limb-unloading samples (*top*). Quantification of Western blot analysis is shown at the *bottom*. Bars represent mean ± standard deviation (*n* = 5 per time point). Protein levels were normalized to α-tubulin loading control (Atrogin-1) or total protein amount where indicated. *P < 0.05; **P < 0.01; ***P < 0.001; ****P < 0.0001 vs. control by one-way ANOVA with Tukey’s multiple comparison test. BW, body weight; mTOR, mammalian target of rapamycin; MuRF, muscle ring finger; MW, molecular weight; p, phospho; Ppar, peroxisome proliferator-activated receptor.

We have recently shown similar expression changes in mitochondrial energy metabolic pathways in a murine hind limb-unloading model of atrophy (depicted in Supplemental Fig. S2*A*) (70). To further explore the temporal changes in the expression of genes in the aforementioned pathways relative to muscle mass loss, an unloading time course experiment was performed. There was a time-dependent loss of muscle mass in this model most evident in muscle groups, such as the soleus, with a high content of slow, oxidative Type I fibers (Fig. 2*A* and Supplemental Fig. S2*B*). Total body weight or food intake did not change significantly over the course of the experiment (Supplemental Fig. S2*C*). We also observed, as expected, an upregulation of *Fbxo32* as early as *day 3* following unloading (Fig. 2*B* and Supplemental Fig. S3). Consistent with the findings with human bed rest, there was a significant reduction in the expression of the major transcriptional regulators of mitochondrial biogenesis and energy metabolism, including PGC-1α, ERRα/γ, and PPARα, as well as an increase in RIP140 (*Nrip1*) (Fig. 2*B* and Supplemental Fig. S3). We also observed a reduction in *Perm1* expression that has previously been shown to be a critical regulator of PGC-1α and ERR in skeletal muscle (Fig. 2*B* and Supplemental Fig. S3) (11). The changes in these transcriptional regulators were coincident with decreased expression of lipid metabolic and mitochondrial genes known to be direct targets for PPAR, ERR, and PGC-1 (Fig. 2*B* and Supplemental Fig. S3). These changes occurred at the earliest time point measured and persisted throughout the course of the study. Importantly, the inhibition of these transcriptional regulators and target genes occurred before a significant decrease in muscle mass (*day 3*), suggesting that a decrease in mitochondrial function contributes to muscle disuse atrophy. These changes in gene expression occurred concurrently with changes in signaling pathways known to be affected by disuse atrophy (Fig. 2*C*).

**Fig. 3.**
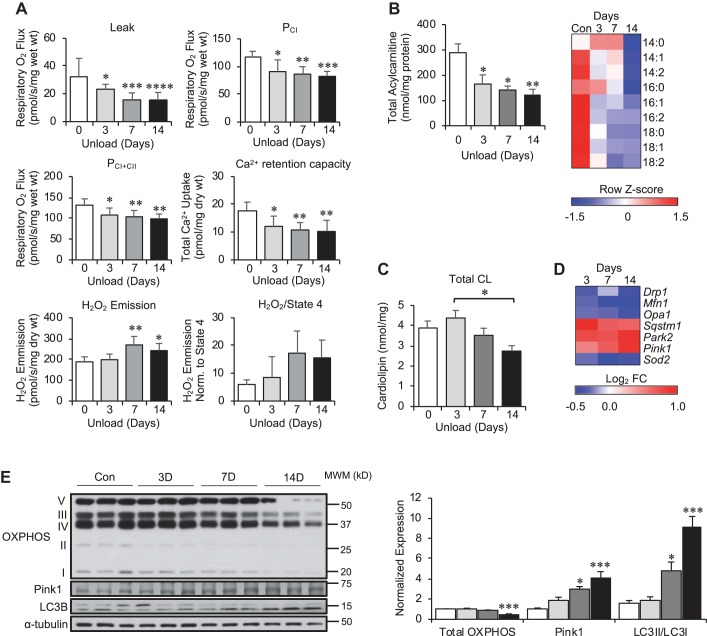
Hind limb-unloading results in reduced mitochondrial content and respiratory capacity. *A*: permeabilized soleus muscle fibers were used to measure proton Leak (Leak), Complex I respiration (P_CI_), Complex II respiration (P_CII_), calcium retention capacity, and H_2_O_2_ emission in control 3, 7, and 14 days following hind limb-unloading samples. *B*: total acylcarnitine levels in soleus are shown for control 3, 7, and 14 days following hind limb-unloading samples. The heat map represents the row Z-score for individual acylcarnitine species in control 3, 7, and 14 days (D) following unloading samples. *C*: total cardiolipin (CL) content in soleus muscle is shown for control 3, 7, and 14 days following hind limb unloading. *D*: the heat map represents the log_2_ fold-change in mRNA levels for the indicated transcript. *E*: Western blot analysis was performed for proteins representative of the electron transport system and oxidative phosphorylation (OXPHOS), Pink1, and LC3B isoforms (*left*). α-tubulin is shown as a loading control. Quantification of Western blot is shown with levels normalized to α-tubulin. Bars represent mean ± standard deviation (*n* = 5 per time point). *P < 0.05; **P < 0.01; ***P < 0.001; ****P < 0.0001 vs. control by one-way ANOVA with Tukey’s multiple comparison test. LC, light chain.

We next sought to determine if the changes that occurred in energy metabolic pathways were reflected in mitochondrial function. Therefore, high resolution respirometry measurements were performed in isolated muscle fibers following hind limb unloading. It should be noted that the assay conditions under which mitochondrial respiration is assessed are not intended to mimic the in vivo conditions of skeletal muscle. However, in our human studies we routinely see correspondence between ex vivo (respiration) and in vivo (^31^P MRS) assays of mitochondrial function (12) (*n* = 22, *r*^2^ = 0.47, *P* = 0.004), indicating that the respiration assays do indeed relate to in vivo conditions of skeletal muscle. As shown in Fig. 3*A*, mitochondrial respiration rates mirrored those of gene expression changes with a significant decrease in complex I and II supported respiration beginning at *day 3* before significant loss of muscle mass. This was accompanied by decreased calcium retention capacity and an increase in H_2_O_2_ emission (Fig. 3*A*). Total acylcarnitine content, reflective of FAO rates, also progressively decreased during the unloading time course (Fig. 3*B*). Levels of specific long-chain acylcarnitines (C14–C18) were significantly decreased as early as 3 days following hind limb unloading (Fig. 3*B* and Supplemental Table S1). Interestingly, the changes in respiration or acylcarnitine levels preceded a change in total mitochondrial content as measured by total cardiolipin and OXPHOS protein content (Fig. 3, *C* and *E*). mRNA levels of autophagy and mitophagy markers, including p62 (*Sqstm1*), *Pink1*, and Parkin (*Park2*), were also significantly increased early during reloading and remained elevated. Likewise, markers of mitochondrial dynamics and reactive oxygen species scavenging were diminished (Fig. 3*D* and Supplemental Fig. S3). Increased protein levels of the Pink1 and the LC3-II/LC3-I ratio also indicated increased rate of mitophagy (Fig. 3*E*). Together, these indicate remarkably early and dynamic mitochondrial remodeling with disuse atrophy that is characterized by diminished oxidative capacity and enhanced mitophagy.

**Fig. 4.**
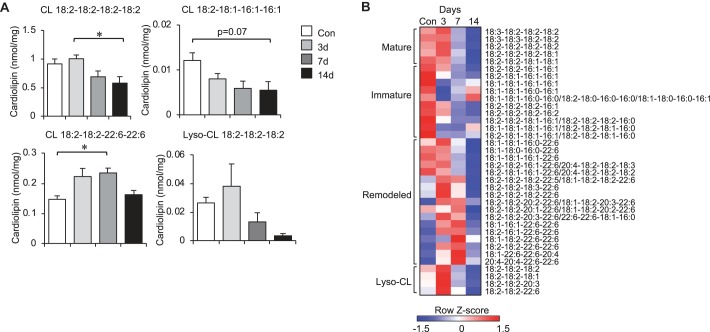
Hind limb-unloading results in marked remodeling of cardiolipin (CL). *A*: levels of the indicated individual CL species in soleus muscle from control (Con) 3, 7, and 14 days (d) of hind limb unloading are shown. Bars represent mean ± standard deviation (*n* = 4 per group). *P < 0.05 vs. control by one-way ANOVA with Tukey’s multiple comparison test. *B*: heat map represents row Z-score of all individual CL species measured. CL species are grouped according to maturation status, including mature, immature, remodeled, and lyso-CL.

Lipidomic analysis of the unloaded muscle was performed to investigate further the dynamic changes occurring during the time course. Although total cardiolipin content decreased during unloading (Fig. 3*C*), examination of individual cardiolipin species revealed a remarkable dynamic remodeling process. By *day 3*, there was a marked elevation of remodeled species containing long-chain and polyunsaturated fatty acid species, such as 22:6, as well as lysocardiolipin levels as the intermediates of cardiolipin remodeling (Fig. 4, *A* and *B* and Supplemental Table S1). However, these levels declined to baseline or even lower by the end of the unloading period on *day 14*. This occurred concomitantly with a progressive decline in mature cardiolipin species, including tetralinoleoyl cardiolipin (18:2–18:2–18:2–18:2), the most abundant species, as well as immature cardiolipin species (e.g., 18:2–18:1–16:1–16:1), indicating a defect of cardiolipin biosynthesis. Analysis of other phospholipid and lipid species revealed minor differences in total content during unloading (Supplemental Table S1).

#### Functional recovery from disuse atrophy is dependent upon proper mitochondrial function.

**Fig. 5.**
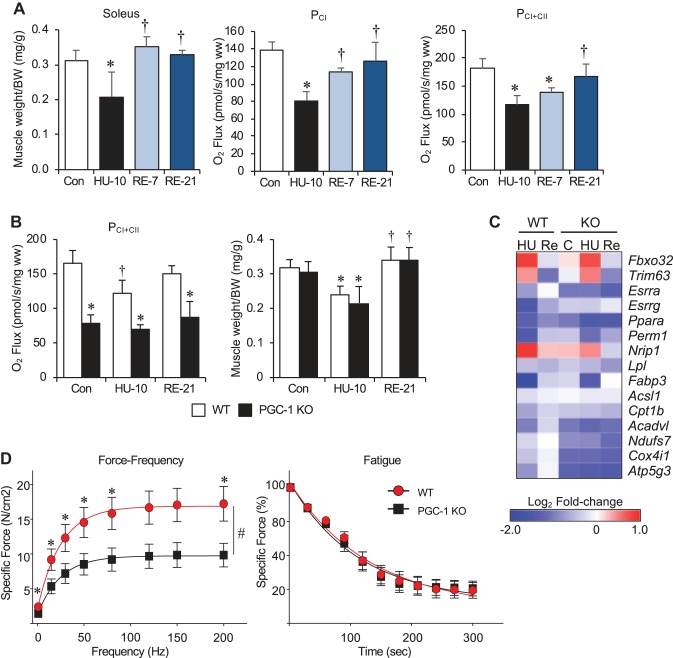
Muscle-specific PGC-1α/β knockout mice display a normal atrophy response and recover mass but not function following reloading (RE). *A*: soleus mass following 10 days of hind limb unloading (HU) followed by 7 and 21 days of RE is shown. Complex I (P_CI_) and Complex I and II (P_CI+CII_) supported respiration from permeabilized soleus muscle fibers is also shown. *B*: Complex I and II (P_CI+CII_) supported respiration (*left*) and muscle mass (*right*) from wild-type (WT) and muscle-specific PGC-1α/β knockout (KO) mice is shown for control, 10 days of HU, and 21 of RE. Bars represent mean ± standard deviation (*n* = 5 per group). *P < 0.05 vs. control; †*P* < 0.05 vs. HU10 by one-way ANOVA with Tukey’s multiple comparison test. *C*: the heat map represents the log_2_ fold-change in mRNA levels for the indicated transcript following 10 days of HU or HU followed by 21 days of RE. Changes are relative to WT control mice. *D*: force-frequency and fatigue protocols were performed with soleus muscle ex vivo from WT and peroxisome proliferator-activated receptor-γ coactivator-1α/β (PGC-1α/β) knockout (KO) mice following 21 days of RE. Points represent mean ± SE (*n* = 8–10 per group). Fatigue is expressed as a percent of WT control at the beginning of the test. *P < 0.05 vs. PGC-1α/β KO by two-way ANOVA with Tukey’s multiple comparison test. #Significant for group × time effect (F) = 15.5; *P* < 0.01. BW, body weight; Ppar, peroxisome proliferator-activated receptor.

These results clearly demonstrated highly dynamic changes to mitochondrial structure, content, and function during unloading and disuse atrophy. However, it is not known how these pathways respond during recovery from disuse atrophy. To this end, WT mice were allowed free ambulatory movement or “reloading” following 10 days of hind limb unloading. Muscle mass recovered rapidly and was completely recovered after 10 days of reloading in all muscle groups examined (Supplemental Fig. S5*A*). This was associated with a marked downregulation of atrogin-1 (*Fbxo32*) and MuRF1 (encoded by *Trim63*) expression as soon as 3 days of reloading (Supplemental Fig. S5*B*). Surprisingly, the expression of many regulators of mitochondrial function and biogenesis, including PGC-1α, ERRα, and PPARδ, were further suppressed after 3 days of reloading (Supplementary Fig. S5*B*). For some genes, including PGC-1α, expression did not fully recover to normal levels and remained inhibited out to 10 days of reloading. Therefore, we extended the reloading period to 7 and 21 days. At 7 days of reloading, soleus mass had completely recovered (Fig. 5*A*). Consistent with the changes in the expression of mitochondrial energy metabolic genes, mitochondrial respiratory capacity measured in permeabilized muscle fibers was markedly depressed after 10 days of hind limb unloading (Fig. 5*A*). However, despite the complete recovery of muscle mass, mitochondrial respiration was still significantly repressed at 7 days of reloading and did not return to control levels until after 21 days reloading (Fig. 5*A*). These data suggest that recovery of mitochondrial function may not be needed for full muscle mass recovery.

To further explore the relationship between mitochondrial function and muscle atrophy, skeletal muscle-specific PGC-1α/β (muscle specific) knockout mice were subject to hind limb unloading (Supplemental Fig. S4). As expected, loss of PGC-1α/β in muscle resulted in severely impaired mitochondrial function (Fig. 5*B*). The mitochondrial respiration capacity in the WT mice decreased with hind limb unloading and recovered with reloading. However, no change in the respiration capacity in the PGC-1α/β mKO mice was noted with unloading or reloading. Remarkably, muscle atrophy in the PGC-1α/β mKO mice was not different from WT following unloading (Fig. 5*B*). The PGC-1α/β mKO mice lost a similar amount of muscle mass as the WT and, surprisingly, recovery of mass was not impaired with reloading. These data were consistent with gene expression analysis that showed no difference in the change in atrophy markers with unloading or reloading but markedly reduced expression of mitochondrial energy metabolic genes in the PGC-1α/β mKO mice (Fig. 5*C* and Supplemental Fig. S6). Remarkably, even in the control WT mice, significant inhibition of PPARα (*Ppara*) expression and target genes, such as CPT-1b (*Cpt1b*) and very-long-chain acyl-CoA dehydrogenase (*Acadvl*), persisted after 21 days of reloading (Fig. 5*C* and Supplemental Fig. S6). Interestingly, ERRα (*Esrra*) expression was reduced in the PGC-1α/β mKO mice across all examined time points, underscoring the autoregulatory regulation between PGC-1α and ERR. Muscle contractile function was also measured in WT and PGC-1α/β mKO mice. As shown in Fig. 5*D*, force production was impaired in the PGC-1α/β mKO mice as compared with WT after 21 days of reloading. Although specific force was lower in the PGC-1α/β mKO mice, the rate of fatigue was not different from WT mice (Fig. 5*D*). This suggests that mitochondrial function is linked with functional recovery of muscle but is discordant with changes in muscle mass following disuse atrophy.

## DISCUSSION

Disuse muscle atrophy is a complex adaptation and often a consequence of chronic illness and/or immobility. We sought to define the transcriptomic changes elicited in elderly subjects following complete bed rest. Strikingly, pathways associated with oxidative metabolism and mitochondria were inhibited, with many of the genes in these pathways predicted to be targets of key regulators of mitochondrial biogenesis and function, including PPAR, ERR, and PGC-1. Very similar changes in gene signatures were also observed in a mouse hind limb-unloading model of muscle atrophy. These changes coincide with reductions in mitochondrial respiratory capacity in isolated muscle fibers. Although the unloaded muscle never approaches maximal respiration, as measured in the ex vivo assays presented here, there are multiple implications for these observations. First, these changes occurred before a significant reduction in muscle mass. In addition, recovery of mitochondrial energy metabolic pathways largely lagged behind mass recovery. Indeed, soleus mass, but not mitochondrial respiration rates, had completely recovered after 7 days of reloading. Collectively, these data suggest that diminished capacity for mitochondrial energy metabolism is a feature that is important for functional recovery of muscle mass following a period of disuse. Finally, our data suggest that the control of mitochondrial function and muscle mass are independently controlled during recovery from a bout of disuse atrophy.

In addition to inhibition of mitochondrial bioenergetic pathways during atrophy, we delineated time-dependent mitochondrial remodeling events. First, total cardiolipin levels decreased proportional to the unloading time. This was driven by decreased concentrations of the major cardiolipin species, including tetralinoleoyl-cardiolipin. Furthermore, there was an early increase in remodeled cardiolipin species containing long-chain polyunsaturated acyl chains (e.g., 22:6) that was evident at 3 and 7 but not 14 days of unloading. These changes coincide with a decrease in mitochondrial respiration rates. It is noteworthy that a decrease in respiration occurs even before a significant decrease in tetralineoyl-cardiolipin. These changes are reminiscent of what occurs in the diabetic heart, in which increases in 22:6 species occur very early in the disease process (26). It is tempting to speculate that these early changes in cardiolipin composition contribute to the impaired respiratory capacity. Interestingly, reductions in mitochondrial respiration and elevations in H_2_O_2_ emission occurred at *days 3* and *7* of unloading, before significant decreases in total cardiolipin or OXPHOS content, suggesting content-independent alterations in mitochondrial energetics. Finally, these changes were accompanied by activation of autophagic and mitophagy markers, including p62 and Pink1/Parkin, at the earliest time examined (Fig. 3).

The diminished expression of ERRα, PPARα, and PGC-1α prior to a significant decrease in muscle mass in the murine hind limb-unloading model suggested that these factors may contribute to the atrophy process. Other studies have also demonstrated a reduction in PGC-1α expression in human muscle atrophy (8, 58). In addition, a protective role for preserved mitochondrial function during muscle atrophy is supported by numerous preclinical studies. Overexpression of PGC-1α or PGC-1β prevents muscle atrophy in various conditions, such as hind limb unloading, denervation, and heart failure (7, 9, 20, 31, 51). Although the precise mechanism is not known, PGC-1 has been shown to inhibit FoxO3 activation of MuRF1 and atrogin-1 expression without affecting protein synthesis pathways (7, 51). These data would imply that the diminished mitochondrial function is a central driver or contributor to the atrophy process. Surprisingly, however, we demonstrate here that loss of PGC-1α/β in skeletal muscle does not impact disuse atrophy. Furthermore, recovery of muscle mass from disuse atrophy was normal in the PGC-1α/β mKO mice.

Although our data did not demonstrate an effect of loss of PGC-1α/β and mitochondrial respiratory capacity on muscle mass, there was a clear effect on contractile function following recovery. This is further supported by our studies with mice lacking PGC-1α/β in muscle. Isolated soleus muscle from PGC-1α/β mKO mice displays impaired force generation and fatigue resistance. Although fatigue in WT mice is diminished with unloading and recovers with reloading, this dynamic is lost in the PGC-1α/β mKO mice. These data are also in agreement with previous work by our group and others showing a severe exercise deficit in mice with loss of PGC-1α/β in muscle (49, 68). This most likely is related to marked deficit in mitochondrial respiratory capacity in the PGC-1α/β mKO mice. Respiratory capacity was markedly lower in PGC-1α/β mKO mice but was not altered during unloading or reloading.

Collectively, our results suggest that mitochondrial function is intrinsically linked to contractile function but not muscle mass. This conclusion is further supported by the observation that muscle mass recovered more quickly than mitochondrial function during reloading (Fig. 5). Consistent with these findings, recovery of muscle function following hind limb unloading is also known to lag behind mass recovery (42). Therefore, we propose that impaired mitochondrial energetics do not directly affect muscle mass but are intrinsically linked with muscle contractile function and are critical for functional recovery. Although the mechanism underlying the lack of functional recovery of muscle in the PGC-1α/β mKO mice is not clear, redox modulation of contractile function may play a role (54).

These results have implications for therapeutic approaches to the recovery of disuse atrophy. Elderly individuals are particularly susceptible to disuse atrophy. Studies have shown that older individuals do not fully recover muscle function (30, 59). The consequences of inadequate recovery from disuse atrophy include increased disability and susceptibility to falls. Although strategies targeting myostatin or its receptor, the activin type IIB receptor, are effective to increase muscle mass in preclinical models and human trials, functional improvement with these agents has been mixed (3, 61). Although force production increases with loss of myostatin signaling, fatigue resistance decreases (21, 39, 45). Interestingly, this has been attributed to impairment in mitochondrial energy production at least in part by loss of PGC-1α signaling (2, 45). Therefore, there is a need for strategies that improve resistance to muscle fatigability in addition to strength or force production. Our data suggest that strategies to improve mitochondrial function and oxidative energy metabolism during the recovery phase could improve muscle recovery following disuse atrophy.

## GRANTS

This work was supported by a grant from the NIH (National Institute on Aging Grant K01-044437 to P. M. Coen and National Institute of Diabetes and Digestive and Kidney Diseases Grant R01-045416 to D. P. Kelly).

## DISCLOSURES

P. M. Coen is a consultant for Astellas/Mitobridge, Incorporated. D. P. Kelly is a consultant for Pfizer, Amgen, and Janssen.

## AUTHOR CONTRIBUTIONS

M.B.T., X.Z., M.P., G.Y., D.P.K., B.H.G., R.B.V., and P.M.C. conceived and designed research; M.B.T., X.Z., R.A.S., M.W., X.H., F.C.G.R., R.B.V., and P.M.C. performed experiments; M.B.T., X.Z., R.A.S., M.W., X.H., F.C.G.R., G.Y., D.P.K., R.B.V., and P.M.C. analyzed data; M.B.T., X.Z., M.W., X.H., F.C.G.R., M.P., G.Y., D.P.K., B.H.G., R.B.V., and P.M.C. interpreted results of experiments; M.B.T., G.Y., B.H.G., R.B.V., and P.M.C. prepared figures; M.B.T., B.H.G., R.B.V., and P.M.C. drafted manuscript; M.B.T., R.A.S., X.H., M.P., G.Y., D.P.K., B.H.G., R.B.V., and P.M.C. edited and revised manuscript; M.B.T., X.Z., R.A.S., M.W., X.H., F.C.G.R., M.P., G.Y., D.P.K., B.H.G., R.B.V., and P.M.C. approved final version of manuscript.
